# Nanomaterial-functionalized biochar hybrids for enhanced Pb^2+^ removal: surface engineering and interfacial mechanisms

**DOI:** 10.1039/d6ra02626e

**Published:** 2026-07-06

**Authors:** Jagpreet Singh, Meenakshi Verma

**Affiliations:** a Faculty of Engineering & Technology, Marwadi University Rajkot-Morbi Road Rajkot 360003 Gujarat India jagpreetnano@gmail.com; b Bahra Research Innovation & Knowledge Cluster (BRIKC), Rayat Bahra University Greater Mohali-140103 Punjab India

## Abstract

The removal of lead (Pb) from wastewater has become essential due to its detrimental effects on human health and on the environmental ecosystem. Moreover, rapid industrialization has led to a significant increase in waste generation, contributing to environmental degradation. These two problems can be addressed through a single sustainable solution: converting waste into biochar. Waste-derived biochar (W-BC) is a promising platform for mitigating Pb^2+^ ions due to its cost-effectiveness, high surface area, and adsorption capacity. Moreover, adsorption potency of W-BC can be enhanced through its functionalization with nanomaterials (NMs). In this regard, the present study delves into recent developments in W-BC for the adsorption of Pb^2+^ ions from wastewater. The comprehensive mechanistic insights into the functionalization of W-BC with different NMs (metal, metal-oxides, carbon, and polymer) are critically discussed, with particular emphasis on the enhancement of surface-active sites and adsorption capacity *via* electrostatic interactions, π–π electron donor–acceptor interactions, hydrogen bonding, surface complexation, and redox processes. This article comprehensively explored the different strategies and favorable experimental parameters (contact time, adsorbent dosage, and pH of solution) for the enhancement of the removal efficiency of W-BC. Additionally, the regeneration approaches for W-BC-based adsorbents have also been discussed, which are crucial for practical and real-time applications. Therefore, the present study provides valuable insights into converting waste materials into an effective lead removal platform for water remediation, thereby contributing to attaining the sustainable development goals (SDGs) and a circular economy.

## Abstract 2

The removal of lead (Pb) from wastewater has become essential due to its detrimental effects on human health and on the environmental ecosystem.

## Graphical abstract

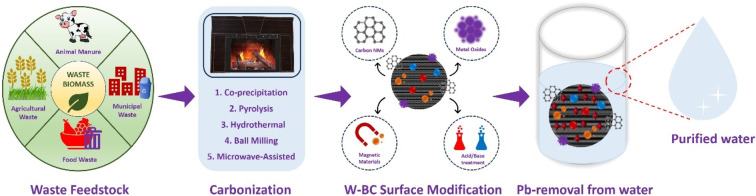

## 1. Introduction

The presence of heavy metals is the biggest threat nowadays due to rapid urbanization, industrial activities, and intensive agricultural practices, causing risks to environmental health.^1^ Heavy metals are trace elements present throughout the earth's crust, their presence in water bodies is highly toxic to humans, animals, and plants because of their toxicity even at very low concentrations.^2^ Lead (Pb^2+^) is found to possess the most significant risks to human health, ecosystems, and agricultural productivity among all the heavy metals present in the environment. It has been reported that more than half of the districts in Punjab have the presence of high levels of Pb^2+^ in their groundwater, which mainly occurs due to the overuse of fertilizers.^3^ For example, Kaur *et al.* evaluated toxic metals in roadway soils near Buddha Nullah in Ludhiana.^4^ Also, the study of Sonkar *et al.*, investigated the pollution status of Pb^2+^ ion in the Sahibzada Ajit Singh (SAS) Nagar district of Punjab, India, through the index of geo-accumulation (Igeo), contamination factors (CF), degree of contamination (DC), pollution load index (PLI), and ecological risk factor (Er).^1^ According to data from the Central Groundwater Board, groundwater in the Punjab districts of Bathinda, Ferozepur, and Muktsar has been found to exceed permissible limits of contamination.^5^ Therefore, Pb^2+^ is considered as a toxic element owing to its high molecular weight and its high varsity among other heavy metals. When compared to other toxic metals, Pb^2+^ accumulates for a longer time period, causing serious damage to human and environmental health. Moreover, the presence of Pb^2+^ in the environment becomes a major cause of water pollution in growing areas.^6–8^ It has been found that people are drinking water contaminated from Pb^2+^ which affects their health badly. Therefore, there is an urgent need to remove pollutants to ensure human life. Moreover, rapid industrialization and urbanization have led to an increase in waste generation, driven by increasing needs of goods and services to fulfil human needs, resulting in a higher rate of waste production.^9^ Several industries, such as pharmaceuticals, textiles, and the food industries release major types of waste that pose a challenge to environmental health.^10^ According to the data from Global Waste Management, the solid waste produced from 2.1 billion tonnes in 2023 and is expected to rise it to 3.8 billion tonnes by 2050.^11^ The best way to manage the growing waste is the preparation of W-BC using this as natural precursor instead of utilizing chemical methods for the removal of Pb^2+^ ions. These two major problems can be addressed with a single solution: converting waste into BC. In recent decades, numerous strategies have been devised for the removal of hazardous metals. This encompasses (i) coagulation, (ii) chemical precipitation, (iii) filtration, (iv) adsorption, (v) ion exchange, *etc*^12–16^. These methods have their own shortcomings that limits their effectiveness in removing Pb^2+^ ions from wastewater. Among several other techniques, adsorption was considered as one of the most widely used removal technologies, attaining significant attention due to its advantages of low cost, straightforward process, and high removal efficiency. Adsorption is a fast removal technique with high potential towards removal of metal ions. Several studies have reported the adsorption method for the effective removal of Pb^2+^ for environmental remediation.^17–19^ There are wide variety of adsorbents available in market which are used for the adsorption of heavy metals for wastewater treatment. Waste-derived biochar (W-BC) is a stable, highly adsorptive black carbon substance that is made in low oxygen environments. Its high surface area, abundance of functional groups, porous structure, and environmental friendliness make it an ideal adsorbent for wastewater treatment. The structure of biochar is distinguished by its high absorption capacity, complex arrangement of carbon rings, and high carbon content, all of which contribute to absorption qualities and stability.^20^ Pristine BC is useful but lacks somehow owing to their several limitations arises due to its hydrophobic surface, small surface area, and fewer available sites to bind and hold the positively charged metal ions. Additionally, unmodified BC has limitations during separation from treated water, making its reuse less efficient. To overcome these challenges, W-BC is often modified with various organic or magnetic materials. Surface functionalization with organic materials enhances the ability of BC to attract and bind metal ions. Furthermore, incorporating magnetic particles into the W-BC allows easy recovery of the adsorbent from aqueous solutions *via* an external magnetic field, which enhances its reusability potential. Currently, numerous reviews have been published summarizing the adsorption of different toxic metals by W-BC in wastewater, but there are very few articles focused on specific heavy metals. For instance, in 2021, Patra *et al.* investigated the preparation and adsorption methods in their study.^21^ In 2022, Berslin *et al.* demonstrated the removal of various pollutants, explaining the different feedstocks and mechanisms used during environmental remediation.^22^ In 2023, Jagadessh *et al.* discussed the adsorption mechanisms for removing pollutants in wastewater treatment. In their study, authors provided insights into the several feedstocks used in the preparation of W-BC, along with a comparison of W-BC adsorbents to others.^23^ In 2024, Dong *et al.* illustrated the W-BC adsorption for targeting pesticides focusing on the mechanisms and functionalization methods of W-BC to enhance the removal efficacy.^24^ These studies often fail to establish a clear relationship between feedstock selection, modification strategies, adsorption mechanisms, and reusability performance. Moreover, inconsistencies in experimental conditions and lack of standardized evaluation criteria limit the comparability of reported results. Table 1 critically compares the present review with previous studies and highlights several limitations in the existing literature. Most earlier reviews discuss biochar adsorption broadly without clearly differentiating feedstock types (*e.g.*, agricultural residues, wood biomass, or fruit wastes), despite their strong influence on surface chemistry and Pb^2+^ adsorption behavior. In addition, experimental conditions and modification parameters are often inconsistently reported, limiting reproducibility and meaningful comparison across studies. Only a few reports, including those by Jagadeesh and Kumkum, provide detailed operational and synthesis information.^23,25^ In contrast, this review provides a focused and critical assessment of Pb^2+^ adsorption using waste-derived biochar (W-BC), emphasizing the role of feedstock composition in governing adsorption performance. The review systematically integrates adsorption mechanisms, surface functionalization strategies, and reusability within a single framework. A major novelty of this work is the comparative evaluation of modification approaches, particularly NM-assisted functionalization, and their influence on Pb^2+^ removal efficiency, selectivity, and stability.

**Table 1:** Comparison summary of key finding of current study with existing literature

Title	Feedstocks	Experimental conditions	Mechanisms	Modifications	Regeneration and reusability of W-BC	Statistical Analysis	Ref.
Remediation of metal pollutants using W-BC	✓	✗	✓	✗	✗	✗	22
Adsorption and functionalization of W-BC	✗	✗	✓	✗	✗	✗	24
Adsorption of pollutants from wastewater	✓	✓	✓	✗	✗	✗	23
W-BC-based adsorption for heavy metal removal	✗	✓	✓	✗	✗	✗	93
W-BC as an adsorbent for Pb(ii) removal	✓	✓	✓	✗	✓	✓	25
Adsorption of potentially toxic elements in water by modified W-BC	✗	✗	✓	✓	✓	✗	94
W-BC derived from fruit by-products using pyrolysis process	✗	✗	✓	✗	✓	✓	95
Waste-mediated W-BC as a sustainable platform for Pb(ii) contamination	✓	✓	✓	✓	✓	✓	This work

Several reviews of biochar-based heavy metal adsorption highlight the same issues. Many focus only on the adsorption performance. This leaves gaps in integrating biochar feedstock, biochar modification strategies, adsorption mechanisms, and biochar applicability. This review is different since it provides a more integrated approach for the study of biochar design strategies and mechanisms for the Pb^2+^ remediation.

The main contributions of this review is the development of the structure–property-mechanism (SPM)-performance framework for the biochar's design to remove Pb^2+^, which integrates the characteristics of the biochar feedstock and the conditions of the biochar production *via* pyrolysis, along the adsorption pathways, and the NM's functionalization. This review also organizes the NM's functionalization in adsorbents into three categories: metal and metal oxide-based systems, carbon-based systems and polymer-based systems. It also reviews the role of each in Pb^2+^ adsorption. Furthermore, this review makes practical assessment of adsorption performance, regeneration capability, and the adsorbent's stability after they have adsorbed Pb^2+^ by integrating them into a single framework. It also provides a research agenda to overcome significant barriers to the practical utilization of the biochar-based Pb^2+^ adsorbents, which include a lack of standardization, regeneration and toxic issues, and the upscaling challenges.

In light of the above discussion, this review presents a comprehensive and critical overview of Pb^2+^ adsorption using waste-derived biochar (W-BC), with particular emphasis on adsorption mechanisms, regeneration efficiency, and reusability toward practical wastewater treatment applications. The review further explores a wide range of biochar functionalization strategies involving NMs and surface modifications, providing clear insight into how these engineered modifications enhance adsorption capacity, selectivity, and stability through distinct physicochemical mechanisms. Unlike previous reports that primarily focus on descriptive summaries, this review systematically evaluates recent advances by correlating feedstock characteristics, synthesis and modification routes, and operational parameters with Pb^2+^ removal performance. Through comparative analysis of existing studies, the review identifies the critical factors governing adsorption efficiency and long-term applicability of W-BC systems. In addition, underexplored waste feedstocks and emerging nanostructured modification approaches are highlighted as promising directions for improving Pb^2+^ remediation. Overall, this review establishes a state-of-the-art understanding of waste-derived biochar-based adsorbents and provides a clear scientific framework for future research, process optimization, and scalable deployment of W-BC technologies for sustainable lead-contaminated wastewater remediation.

## 2. Synthesis of W-BC

### 2.1 Overview of different synthesis methods

**Fig. 1:**
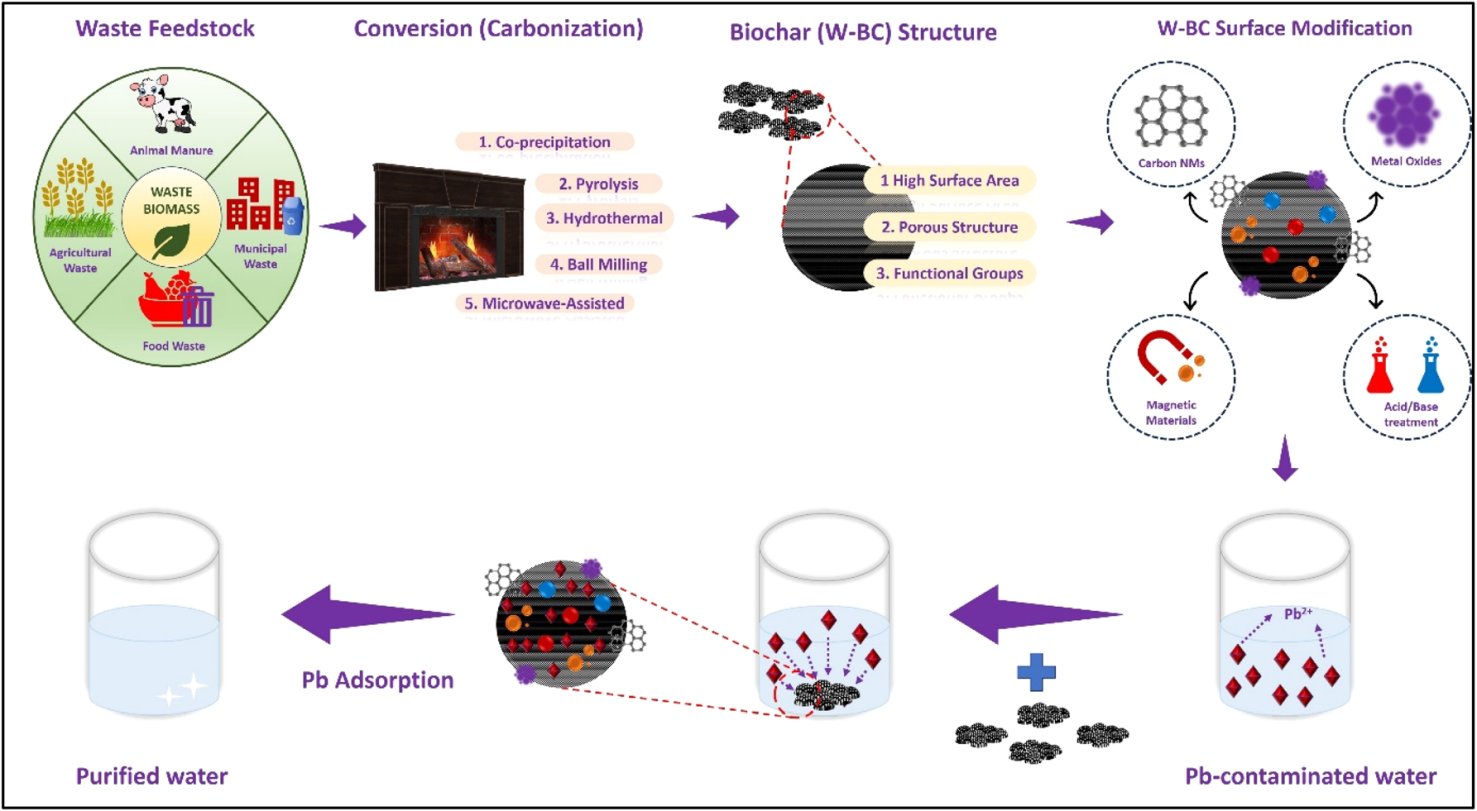
Overview of preparation of W-BC derived from different feedstocks for wastewater treatment.

W-BC is a carbon form produced mainly using pyrolysis of organic materials, primarily biomass, which have diverse applications, including,carbon sequestration, soil amendment, and wastewater treatment.^26–35^ The synthesis of W-BC can be approached through several methods, each with distinct characteristics, advantages, and limitations. The different properties of W-BC vary with their synthesis techniques based on the different feedstocks utilized and different parameters given during the preparation of W-BC.^26,36^ All the synthesis techniques have their own advantages such as W-BC yield, their sorption capacity, and limitations. The method's choice depends on the final product's desired physicochemical properties. There are different parameters including during the preparation of W-BC including the temperature we provided to different methods. Fig. 1 represents overview of different feedstocks utilized in preparation of W-BC. The nature of feedstock, pyrolysis conditions (temperature, heating rate, residence time), and post-treatment modification techniques all have a significant impact on the physicochemical characteristics of W-BC. The absence of defined synthesis conditions is a significant constraint in the literature despite a great deal of study, which makes it difficult to compare reported adsorption performances directly. To provide a better understanding of the synthesis techniques of W-BC, a comparison is presented Table 2. This comparison highlights each method's significant advantages, disadvantages, and various parameters for a better comparison with a broad understanding.

**Table 2:** Overview of synthesis of W-BC: a comparison among natural precursors, process conditions, advantages, disadvantages, and applications

Sr. No.	Method	Precursor	Parameters	Advantages	Disadvantages	Applications	References
1	Pyrolysis	Kiwi branch, rice husk, sawdust, maize straw, jackfruit seed	300–700 °C	Simple operation, high yield, more stability	Requires high temperature, gaseous pollutants release	Carbon sequestration, water treatment	51, 80, 87, 96 and 97
2	Microwave-assisted pyrolysis	Banana peels, crop straws	300–700 °C	Rapid heating, less solid residue	High energy consumption, low yield	Waste treatment, pollutant removal	98 and 99
3	Hydrothermal carbonization	Rice husk, pamelo fruit leave	180–300 °C	High carbon-content, produces more stable W-BC	Time-consuming, requires high-pressure reactors	Waste management, energy storage	39 and 54
4	Ball-milling	Bamboo powder, bone	20–25 °C	High efficiency, low-cost	Limited feedstock types, structural damage	Energy storage, catalysis	100 and 101
5	Gasification	Cotton stalk, larch wood chips	750–900 °C	High energy recovery, cleaner process	Low yield, expensive equipment	Energy production, soil amendment	40 and 102
6	Co-precipitation	Corn straw	20–25 °C	Cost-effective, high adsorption capacity	Agglomeration, instability	Carbon sequestration, environmental remediation	63

### 2.2 Raw materials

The feedstock type plays a crucial role to determine the structural and functional properties of W-BC. The waste biomass can be categorized into agricultural, forestry, animal, municipal, and food-based residues. However, a critical evaluation reveals that most studies focus on availability rather than systematic performance-based selection, which remains an unresolved research gap.

#### 2.2.1. Agricultural waste

Agricultural waste is a significant yet underused resource for the preparation of W-BC, providing a sustainable alternative to conventional feedstocks like wood. The heavy use of agricultural waste generates a high amount of biomass waste including crop residues, husks, and leaves transforming these materials into W-BC. Agricultural waste is a significant component of lignocellulosic biomass present on the surface of the Earth, representing an abundant and underutilized resource for the preparation of W-BC. Lignocellulosic biomass primarily consists of constituents of cellulose, hemicellulose, and lignin.^37^ These components are found in various residues produced during agricultural activities, such as rice husks, corn stalks, straw, and other plant materials left after harvest. These materials are often burned immediately after agricultural activities, which produces air pollution. We can utilize this agricultural waste to enhance soil health and support water remediation by converting this agricultural waste into W-BC. The conversion of biomass into W-BC forms a stable, carbon-rich product that reduces agricultural waste, thereby supporting environmental sustainability. The properties of W-BC vary according to the specific agricultural feedstock and the preparation method. Furthermore, utilizing agricultural waste for W-BC production not only minimizes waste but also contributes to a mitigation greenhouse gas emissions and, fostering sustainability by turning waste into a resource. The main limitation observed was that adsorption performance is mainly attributed solely to feedstock type without investigating the effect of pyrolysis conditions. For instance, In 2021, Deng *et al.* investigated modified W-BC prepared using corncob as natural precursor to enhance the adsorption of Pb^2+^ ions.^38^ In 2022, Li *et al.*, developed amine-functionalized W-BC used for treatment of wastewater *via* adsorbing Pb^2+^ ions.^39^ In 2021, Gao *et al.* synthesized W-BC derived from agricultural waste cotton stalk to remove Pb^2+^ ions.^40^ Also, Ahmed *et al.* developed modified W-BC using watermelon seeds to enhance the adsorption of Pb^2+^ ions.^41^

#### 2.2.2. Forestry waste

According to the UN FAO, 10 million hectares (ha) forests are cut down yearly, mostly due to rapid industrialization.^42^ This alarming rate of deforestation seriously impacts biodiversity, climatic stability, and the economic viability of forest-dependent communities. Several types of forestry waste, such as branches, leaves, sawdust, and residues from rain trees and krachid trees, are often disposed of improperly, contributing to environmental pollution. These types of waste release greenhouse gases that affect the climate. Additionally, burning these wastes to clear land releases smoke into the air, affecting air quality and human health. These residues can be used for the production of W-BC and can be utilized in erosion control and landscaping. Proper management or disposal of forestry waste promotes sustainable forestry by minimizing environmental impacts and enhancing ecosystem health. For instance, in 2022, Cheng *et al.* developed MgO-modified W-BC derived from crofton weed to boost the adsorption potency of Pb^2+^ ions. The proper disposal of forestry waste promotes a sustainable environment and reduces the harmful impact of Pb^2+^ ions.^43^

#### 2.2.3. Animal manure

Animal manure is being increasingly accepted as a viable feedstock for the production of W-BC. This approach involves pyrolysis of organic materials under high temperature conditions, typically from livestock, to convert it into a stable carbon-rich product. The different properties of W-BC vary according to the different physical and chemical properties, allowing for the diversity of their applications, such as adsorbents for pollutant removal and other applications.^44^ Animal manure not only addresses waste management problems but also improves crop yield by enhancing the fertility of soil, especially for nutrient-deficient soils. Animal manure provides essential nutrients such as nitrogen, potassium, and phosphorus, which enhance water retention and are beneficial for microbial activity. For instance, Ye *et al.* 2022 developed W-BC prepared from cattle manure for the efficient adsorption of Pb^2+^ ions. In their study, the authors compared the adsorption capacities of W-BC derived from cattle manure and cherry wood.^45^ The results demonstrated that the W-BC synthesized from cattle manure exhibited higher yield and sorption capacity compared to that derived from cherry wood, likely due to its higher mineral content.

#### 2.2.4. Municipal waste

The W-BC prepared from municipal waste, particularly from sewage sludge used as a byproduct for waste management and addressing environmental challenges. Sewage sludge is rich in essential nutrients and organic matter used to prepare W-BC under the pyrolysis process. The conversion of W-BC from sewage sludge not only improves soil fertility but also reduces the volume of sewage sludge which contributes to pollution. Approximately, 2.1 billion tonnes of solid waste generation is estimated in 2023 and it is likely to grow to 3.8 billion tonnes by 2050. The direct waste management costs high approx. USD 252 billion in 2020, which rises to 361 billion due to poor health conditions, and climate change. There is an urgent need of waste management in 2050 with the annual cost could almost double to a staggering USD 640.3 billion.^11^ This study intends to provide a global accounting of waste generation and management patterns to fill the gap.

#### 2.2.5. Food waste

Food waste is a significant global issue, with millions of tons discarded each year, often ending up in landfills where it decomposes and emits greenhouse gases. According to the report of the United Nations Environment Programme, about 68.7 million tons of food is wasted each year in Indian homes.^46^ To address such issues, W-BC is prepared from food waste *via* pyrolysis in the absence of oxygen to reduce the waste. Utilizing food waste for W-BC production aligns with circular economy principles, turning a waste product into a valuable resource and contributing to sustainable agriculture and environmental stewardship. Food waste includes fruit and vegetable peels, eggshells, cooked food residues, tea leaves, and coffee grounds.^47^ There are types of foods in which waste product is utilized as precursors during the W-BC formation process. For instance, in 2022, Li *et al.* investigated amine-functionalized MgFe_2_O_4_-BC to enhance the adsorption efficacy of Pb^2+^ ions. The study found that ion exchange, complexation, pore filling, and electrostatic attraction were the primary mechanisms responsible for Pb^2+^ adsorption. The doping of MgFe_2_O_4_–NH_2_ onto the BC surface introduced to diverse adsorption sites for Pb^2+^, resulting in significantly higher adsorption capacity compared to bare BC.^39^ The main gap in the ongoing research is the absence of standardized synthesis protocols and unified evaluation criteria, which leads to inconsistent adsorption performance reporting across studies. Thus, the development of controlled comparative studies and the establishment of performance benchmarking systems for W-BC materials should be the main goals of future research. Overall, the physicochemical features of W-BC are directly influenced by the synthesis process and feedstock type, such as surface area, porosity, mineral content, and functional group distribution. These parameters determine the major adsorption mechanisms, which include ion exchange, electrostatic attraction, surface complexation, and precipitation. As a result, knowing the synthesis-structure–property link is critical for rational design of biochar-based adsorbents and meaningful comparison across studies.

The Pb^2+^ adsorption mechanisms of W-BC are controlled by biochar surface reactivity, its functional groups, and mineral phases. These are determined by the synthesis conditions of the biochar. Before Pb^2+^ adsorption mechanisms can be understood, the effects of biochar synthesis conditions on its physiochemical properties and functional groups must be elucidated.

## 3. Functionalization of biochar with different nanostructured materials for Pb(ii) removal

For systematic clarity, we group the primary Pb^2+^ adsorption mechanisms into five main types: electrostatic attraction, ion exchange, surface complexation, precipitation, and redox-assisted immobilization. This provides the opportunity to directly analyze various biochar systems and constructs a SPM relation.

Further, the adsorption mechanisms presented in the following sections stem directly from the physicochemical properties that arise following biochar synthesis and surface modification. Subsequent changes in feedstock composition along with the pyrolysis conditions, and the nature of functionalized NMs, can alter the shape distribution, active sites' density of mineral constituents, surface chemistry, and porosity of the final biochar. These properties will ultimately governs the most dominant pathways of Pb^2+^ immobilization.

The effectiveness of W-BC can be efficiently explored using a SPM concept. Surface functionalization with nanostructured materials alters the physicochemical properties of W-BC, affecting adsorption pathways and removal efficiency of Pb^2+^ ions. W-BC has gained significant attention from researchers owing to its high efficiency as an adsorbent for the removal of lead. Pb^2+^ ion's adsorption onto the surface of W-BC is a surface-mediated process similar to heterogeneous catalytic adsorption, where Pb^2+^ ions interact with functional groups present on the nano-structured carbon surface. The porous structure of W-BC act as reactive platform, where Pb^2+^ ions undergoes adsorption process, such as ion exchange, electrostatic attraction, and chemical interactions, can remove contaminants from aqueous solution. Adsorption involves two types: physical adsorption, which involves the weak van der Waals forces, and chemical adsorption, which includes strong interaction between the functional groups and metal ions. The numerous studies have reported the utilization of biochar for lead removal. For instance, in 2024, Liu *et al.* investigated W-BC derived from rice straw at different pyrolysis temperatures to assess its adsorption efficacy. The study found ion-exchange and precipitation as the dominant mechanisms for Pb^2+^ removal. The presence of minerals on the BC surface led to the precipitation of Pb^2+^ ions, while the distribution of carbon and oxygen-containing functional groups affect the binding energy and adsorption capacity.^48^

Likewise, Latifiana *et al.* investigated the preparation of BC from sawdust for the effective removal of Pb^2+^ ions. The optimum pH of solution selected for study is 5.7, where electrostatic attraction between the positively charged metal ions and negatively charged species present on the surface of BC enhanced adsorption. The presence of surface functional groups on nano-porous structure of biochar enabled binding of metal ions *via* chemical interactions. The precipitation also occurred due to the formation of OH^−^ metal complexes with heavy metals which enhance the removal potential of BC.^49^ Also, In 2021, Gao *et al.* developed BC from cotton stalks through a pyrolysis process for the effective adsorption of Pb^2+^ ions. The study explained the removal of Pb^2+^ ions through a physical adsorption mechanism. The study detailed how precipitation occurred on the BC surface as Pb^2+^ ions entered the pores. Furthermore, ion-exchange and complex formation play the main role, where Pb^2+^ ions interacted with functional groups present on the surface of biochar.^40^

In 2021, Cheng *et al.* investigated the adsorption of Pb^2+^ ions using BC. The study optimized the pyrolysis temperature to examine its effect of temperature on adsorption. The study found that at temperatures between 600-700 °C, adsorption was highest due to increased BET surface area, which provided more adsorption sites. Due to presence of hydroxyl, carboxyl groups on the adsorbent surface, further increase the adsorption of Pb^2+^ ion. However, as the temperature rises to 800 °C, the structure of BC destroyed and hence decrease in efficacy observed. The study demonstrated that chemical sorption was the primary mechanism for the adsorption of Pb^2+^. Additionally, the study revealed that metal ions such as K^+^, Ca^2+^ and Mg^2+^, undergo ion exchange during the adsorption process.^50^ Similarly, Li *et al.* prepared BC from sewage sludge and modified with the silica extracted from rice husk for the preparation of green cost-effective W-BC composite. The composite came out to be best adsorbent for the removal of Pb^2+^ due to its environmental friendliness and cost-effectiveness. The modification of W-BC with silica has been done to enhance the adsorption efficacy of W-BC. The study reported the 6.43 times increase in adsorption capacity compared to the bare W-BC.

Ion-exchange and electrostatic adsorption are the main mechanisms used to explain the adsorption of Pb^2+^ ion.^51^ For instance, in 2024, Ning *et al.* explored the adsorption of Pb^2+^ on the surface of BC using a co-pyrolysis composite of water treatment residue and rice straw. The study reported an increase in the BET surface area of WTR-BC composite of 46.73 m^2^ g^−1^ as compared to bare BC, which had a surface area of 15.11 m^2^ g^−1^. This enhancement in surface area significantly increased the number of active sites and functional groups on the adsorbent surface, thereby improving the binding of Pb^2+^ through electrostatic interactions and surface coordination. The removal capacity of Pb^2+^ was found to increase with the increase in pH of the solution. At low pH, limited deprotonation of oxygen-containing functional groups results in weaker electrostatic attraction between Pb^2+^ ions and the biochar surface. In contrast, at high pH values, the removal capacity increases due to a reduction in competition for adsorption sites.^52^

In another study, Zhou *et al.* prepared W-BC modified with potassium phosphate using a pyrolysis approach in the absence of oxygen. The study optimized the temperature and concentration like experimental parameters of potassium phosphate to achieve the highest adsorption capacity values. In this study, complexation occurs between the oxygen-containing functional groups which are present on the carbon surface.^53^ In 2022, Dinh and his coworkers investigated the adsorption of Pb^2+^ using BC derived from pomelo fruit peel. The study optimized Pb^2+^ concentrations and found that the adsorption capacity increased significantly with higher Pb^2+^ concentrations, rising from 133 mg L^−1^ to 389 mg L^−1^, while the adsorbent dose remained constant, ensuring the no effect on the availability of adsorption sites. The adsorption mechanism was attributed to Pb^2+^ ions adsorbed onto the porous structure, the transfer of metal ions, and interactions between the surface area of BC and active sites. The study primarily followed ion-exchange and electrostatic interactions during the adsorption stages.^54^ Initially, an increase in pH altered the charge on the functional groups of BC, enhancing electrostatic attraction between the positively charged Pb^2+^ ions and the negatively charged functional groups on the BC surface. Subsequently, Pb^2+^ ions diffused into structural defects within the BC, where they interacted through ion-exchange mechanisms. The study optimized BC performance for the effective removal of Pb^2+^ ion.^55^

However, pristine biochar limits its adsorption efficiency due to low availability of functional groups, surface reactivity, and low regeneration capacity. To overcome such issues, different types of nanostructured materials are employed for functionalization of BC. The surface modification of BC with NMs enhances the adsorption capacity by improving the physicochemical properties of BC. The incorporation of metal, metal-oxide based, carbon-based, and polymer-based NMs improves the porosity, density of functional groups, and availability of active sites of W-BC. Consequently, functionalized W-BC exhibit superior adsorption capacity and improved stability as compared to pristine biochar.

The mechanistic insights revealed that adsorption of Pb^2+^ onto the biochar surface is controlled by chemical interactions (ion exchange, complexation, precipitation, and coordination bonding) and physical adsorption (pore filling, electrostatic attraction). However, the relative contribution of these pathways varies widely and is highly dependent on the type of NMs modification, surface functional groups, and solution chemistry, all of which are not sufficiently standardized across studies.

The majority of studies offers improved adsorption performance but do not clearly demonstrate a SPM relationship, which makes cross-comparison challenging. This is a significant shortcoming of the current literature. Therefore, to comprehend functionalization effects, a more systematic classification based on nanomaterial type is required.

### 3.1 Metal and metal-oxide-based NMs

Metal and metal-oxide-based NMs have been widely investigated for the functionalization of W-BC due to their ability to enhance adsorption performance through surface modifications and catalytic activity. The MgO, Fe_3_O_4_, MnO_2_, and ZnO improves the Pb^2+^ removal through several mechanisms. The incorporation of metal oxides onto the surface of W-BC increases the active binding sites, surface reactivity, and electron transfer capability. A common feature of metal oxide modification is the generation of surface hydroxyl (–OH) groups, which act as active binding sites for Pb^2+^ ions and promote electrostatic attraction and surface complexation. In addition, these modifications enhance surface area and porosity, thereby improving diffusion and adsorption capacity.

The metal oxides like ZnO, MgO increases the solution pH due to partial dissolution which facilitates formation of metal hydroxides, thereby enhancing adsorption *via* surface precipitation. In contrast, transition metal oxides such as Fe_3_O_4_, MnO_2_ contributes to redox active properties. Among various metal oxides, MgO and ZnO primarily improve adsorption *via* precipitation mechanisms and alkaline surface behavior. Their dissolution raises the pH of the solution, encouraging the production of Pb(OH)_2_ and increasing the effectiveness of removal. Fe_3_O_4_-based composites offer further benefits because of their strong ion-exchange capacity and magnetic characteristics, which facilitate simple separation and effective adsorption over a wide pH range. Because of their varied oxidation states, which improve inner-sphere complexation and surface reactivity, MnO_2_-based systems contribute both adsorption and redox activity.

For instance, lisowaski *et al.* investigated the dual functionality of W-BC with metal oxide *via* photocatalytic degradation and selective oxidation.^56^ In another study, Zhou *et al.* illustrates the generation of reactive oxygen species, explaining the catalytic mechanism using hydrochar and pyrochar.^57^ In 2025, Mudassar *et al.* prepared a ZnO/biochar nanocomposite for Pb^2+^ removal from wastewater. The incorporation of ZnO NPs induces a rough, porous surface with abundant active adsorption sites, leading to a Pb^2+^ removal efficiency of 90.3%.^58^ Similarly, Wan *et al.* have synthesized the biochar using peanut shell-supported hydrated manganese oxide for the removal of lead. The authors have reported that hydrated manganese oxide (HMO) NPs show higher adsorption capacity than bare BC.

**Fig. 2:**
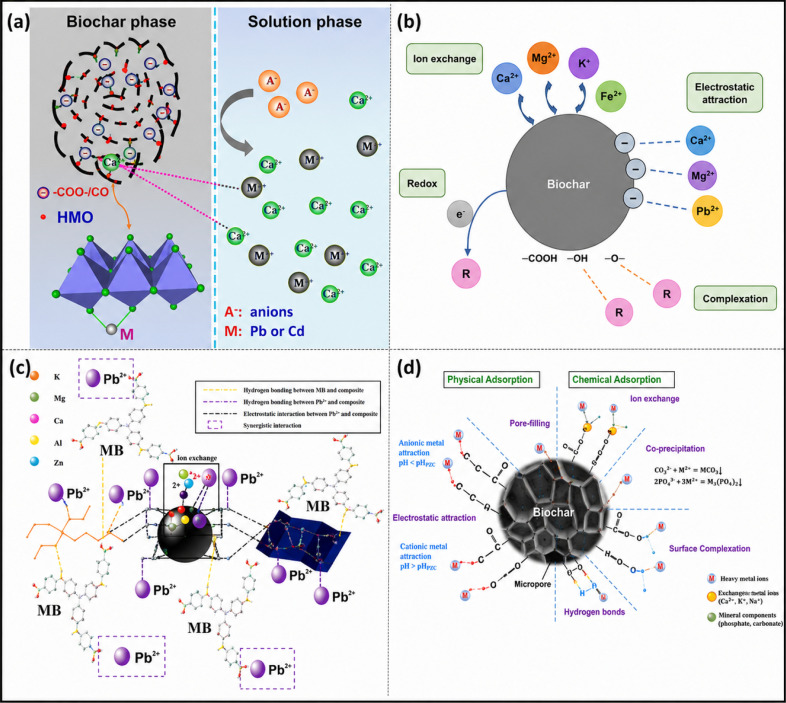
(a) Proposed mechanism of hydrous manganese oxide-modified biochar (HMO-BC) for the removal of lead (reproduced from ref. 59 with permission from Elsevier copyright 2018); (b) adsorption of Pb^2+^ ions onto the surface of biochar/nano-FeS and starch/chitosan composites (originally drawn by authors); (c) adsorption mechanism of ATES/BC/MXene for the removal of Pb^2+^ ions (reproduced from ref. 67 with permission from Elsevier copyright 2024); (d)Schematic representation of adsorption mechanisms in removal of lead using W-BC (reproduced from ref. 69 with permission from Elsevier copyright 2024).

Moreover, the oxygen containing functional moieties on the surface of W-BC provides negative charged surface that facilitate electrostatic attraction of metal cations within the porous matrix. Consequently, the Donnan membrane effect was observed, which attracts positively charged metal ions into the pore channels of the adsorbent. Subsequently, the metal ions form a chemical bond with the surface atoms, as HMO provides binding sites for metal ions through inner-sphere surface complexation mechanisms. Furthermore, as per structural investigations like Extended X-ray Absorption Fine Structure (EXAFS), Pb^2+^ ions primarily attach to manganese oxides by double-corner-sharing complexation at particle edges with visible hydroxyl groups and triple-corner-sharing complexation at interlayer vacancies as shown in Fig. 2a.

As a result, the integrated system efficiently sequesters toxic metal ions from aqueous environments by an associated mechanism that involves electrostatic attraction, Donnan membrane-assisted diffusion, and particular surface complexation.^59^ In 2023, the study by Singh *et al.*, investigated the adsorption of Pb^2+^ ions using a magnetic BC composite derived from waste pine needles. In this study, the authors doped the magnetite (Fe_3_O_4_) nanoparticles into W-BC to prepare 3-aminopropyl triethoxysilane-functionalized magnetic BC (MBC). The magnetic NPs are connected to W-BC *via* hydrogen bonding. This study focused on the metal exchange of ions and interaction with the W-BC functional groups. During the process, metal ions reduced the oxygen *via* cation exchange with the magnetic nanoparticles embedded in the W-BC structure.^60^ In 2021, Deng *et al.* prepared magnesium-modified W-BC for Pb^2+^ ion removal in wastewater treatment. The study demonstrated enhanced properties of BC through surface modification with Mg (OH)_2_ and MgO particles. The mechanisms underlying the adsorption process were explained by surface precipitation, ion-exchange, and complexation. Modification significantly improved the porosity of BC, increasing its surface area from 12.68 m^2^ g^−1^ (pristine BC) to 52.41–174.29 m^2^ g^−1^ for Mg-modified BC (Mg-BC). The study also highlighted that the enhanced metal ion adsorption by 15% Mg-BC was attributed to the increased pH of the solution caused by Mg(OH)_2_ formation.^38^

In a study by Zhou *et al.* magnetic BC was prepared by activating macadamia nutshells, which were pretreated with FeCl_2_. The BC exhibited a highly porous structure, confirming pore-filling as a mechanism of adsorption. The surface of the adsorbent becomes negatively charged at pH 6, where the electrostatic attractions are more dominant due to strong interaction between positively charged Pb^2+^ and negatively charged surface. The study further revealed that the BC surface contained numerous functional groups, which interacted with Pb^2+^ ions through complexation, enhancing the removal efficiency.^61^ The other study demonstrates the effectiveness of magnesium oxide loaded BC composite in the removal of Pb^2+^. The study reported the removal mechanism based on the generation of hydroxyl ions that form complex with metal ions to form insoluble hydroxides. Additionally, the cation exchange between metal ion and Mg^2+^ ion contributes to removal of metal ion. The synthesized MBC achieved high adsorption capacities of 829.11 mg g^−1^ for Pb^2+^ attributed to a combination of precipitation, ion-exchange, cation–π interactions, and functional group complexation.^62^ The increase in surface hydroxyl groups, which act as active binding sites for Pb^2+^ ions and enhance electrostatic attraction and surface complexation, is a characteristic of all metal oxide modifications. Furthermore, increased surface area and porosity promote adsorption and diffusion. In comparison, precipitation dominance makes MgO-modified biochar most successful under neutral to slightly acidic conditions, whereas redox-active behavior makes Fe- and Mn-based systems more widely applicable. ZnO-based compounds help by increasing surface area and providing photocatalytic support when exposed to light.

With every aspect considered, the combination of metal oxides and biochar results in a multipurpose adsorbent system where surface precipitation, ion exchange, adsorption, and AOP-assisted redox processes cooperate to effectively remove Pb^2+^. Metal oxide functionalization improves adsorption efficiency by increasing the density of surface hydroxyl (-OH) groups, which serve as active binding sites for Pb^2+^ through electrostatic attraction and surface complexation. Additionally, increased surface area and porosity promote mass transfer and diffusion of Pb^2+^. Fe- and Mn-based systems have broader applicability due to their redox-active activity, providing multiple paths for Pb^2+^ immobilization. ZnO-based composites also help by increasing surface area and, in certain instances, enabling photocatalytic support under light irradiation.

Despite these advantages, limitations such as nanoparticle aggregation, lower stability in acidic environments, and potential metal leaching must be addressed. Metal oxides combined with biochar form a multifunctional adsorbent system that effectively removes Pb^2+^ through surface precipitation, ion exchange, adsorption, and redox reactions.

### 3.2 Polymer-based NMs

The functionalization of biochar with polymer-based NMs involves the incorporation of chitosan, alginate, polyaniline, polypyrrole, and polyacrylic acid. Several studies have reported the incorporation of polymer-based NMs to enhance the adsorption efficiency and stability of W-BC. For instance, Liang *et al.* investigated the W-BC from corn straw doped with CaFe-layered double hydroxide for the adsorption of Pb^2+^ ions. The analysis illustrated that CaFe-LDH@CSB was loaded with LDH, which enhances the functional groups on the surface of the adsorbent for the removal of metal ions. Moreover, authors states that CaFe-LDH@CSB was negatively charged, which attracts the positively charged metal ion onto its surface following electrostatic attraction. Furthermore, surface precipitation, ion-exchange, and complexation are the proposed mechanisms for the adsorption of toxic metals on adsorbent CaFe-LDH@CSB.^63^ In 2022, Bak *et al.* developed W-BC from agricultural waste modified with chitosan to enhance the adsorption of Pb^2+^ ions. The study observed possible interactions between the pollutant and the adsorbent surface facilitated by the presence of numerous groups on the surface of W-BC. The adsorption of metal ions occurred through the surface precipitation of sparingly soluble hydroxides.^64^ In 2022, Wang *et al.* prepared the biochar/nano-FeS and starch/chitosan composites for the adsorption of Pb^2+^ ions. The study follows the complexation of Pb^2+^ ions with functional groups present on the BC surface. Additionally, the electrostatic forces between the negatively charged anions on the BC surface and the positively charged cations played a significant role in enhancing adsorption efficiency. The study reveals that the carbon-oxygen double bond of the –CO group opened to form a free radical that undergoes electrostatic interactions with the positively charged ions as shown in Fig. 2b.^65^ The Fig. 2b demonstrates the occurrence of multiple mechanisms on the surface of FeS/starch/chitosan-modified biochar system. Ion exchange, electrostatic attraction, redox interaction, and surface complexation are the main mechanisms influencing the adsorption process. FeS facilitates additional binding affinity and redox processes, whereas functional groups like –OH and –COOH on the composite surface interact with Pb^2+^ ions. While ion exchange takes place between Pb^2+^ and intrinsic cations (such as Ca^2+^ and Mg^2+^), the negatively charged surface increases electrostatic attraction. Increased adsorption performance is result of these combined reactions.

The study by Liu *et al.*, investigated the adsorption of Pb^2+^ ions using various W-BC composites, nano iron sulfide@walnut shell BC (FeS@WNS), Starch-FeS@WNS, and Chitosan FeS@WNS, to evaluate the adsorption efficacy of Pb^2+^ ions. The incorporation of FeS enhances the removal efficiency of Pb^2+^ ions, and the presence of starch significantly improves the stability and reduces the problem of agglomeration. The proposed mechanism for Pb^2+^ removal primarily involves electrostatic attraction. Furthermore, the functional groups present on the surface of BC undergo protonation, which makes the surface of W-BC negatively charged and interacts with the positively charged Pb^2+^ ions. Moreover, FeS in the composite interacts with Pb^2+^, and form insoluble PbS precipitates, further contributing to Pb^2+^ ion removal.^66^

### 3.3 Carbon-based NMs

Carbon-based NMs have attracted attention for the functionalization of W-BC due to their exceptional surface properties. These type of NMs provide active binding sites and facilitate stronger interaction towards the removal of lead ion. For instance, Ijaz *et al.* developed an aminopropyltriethoxysilane (APTES)-functionalized BC/MXene composite to enhance the adsorption capacity for Pb^2+^ ions. According to the study, the effective adsorption of lead ions from wastewater was attributed to the electrostatic interactions, surface coordination and hydrogen bonding formed between the composite adsorbent materials and lead ions. The surface chemistry and the rate of adsorption were synergistically strengthened by the hybrid nanostructure. Furthermore, the adsorption capacity of Pb^2+^ ions increases after grafting aminopropyltriethoxysilane functionalized biochar on the MXene. Simultaneously, the MXene support enhances surface area and electron transfer capability, strengthening adsorption interactions. The hybrid structure promotes strong coordination between Pb^2+^ ions and functional groups, leading to enhanced adsorption capacity as shown in Fig. 2c.^67^ In another study, Liu *et al.* prepared biochar-supported carbon nanotube and graphene NCs for the detection of lead. The study found that enhanced adsorption capacity was attributed to the incorporation of carbon NMs (CNT and GO) into the biochar. When compared to bare biochar, the sorption capacity of carbon-based NMs modified NCs enhances 2–5 times for Pb(ii). The authors found that the presence of oxygen-containing functional groups such as carboxyl (–COOH) and hydroxyl (–OH), on graphene oxide and functionalized Carbon nanotubes, facilitates strong surface complexation with metal ions in aqueous systems.^68^

Overall, the adsorption of Pb^2+^ onto biochar surface involves ion-exchange, surface complexation, electrostatic attraction, coordination and precipitation mechanisms. The nanostructured carbon matrix serves as a reactive platform that facilitates surface-mediated immobilization of metal ions in heterogeneous catalytic surface processes, which are comparable to these mechanisms. The high density of functional group on the biochar surface also enhances its porosity which further promotes its reactivity. This demonstrates the effectiveness of biochar towards the removal of lead in the context of catalytically analogous surface interactions. Fig. 2d^69^ summarizes the general adsorption mechanisms of Pb^2+^ on biochar including physical and chemical processes.^69^ Physical adsorption includes pore-filling and electrostatics adsorption, while chemical adsorption involves ion-exchange, surface complexing, coprecipitation, and hydrogen bonding. However, the dominance of the above processes depends on conditions like pH value, surface functional groups, and presence of minerals. Biochar can be considered as multifunctional adsorbents that utilize their porous structure and surface chemistry for removing Pb^2+^ ions. Several studies have worked on the wastewater treatment by utilizing different preparation techniques with different experimental conditions shown in Table 3.

**Table 3:** Summary of chemical, physical, and mechanical properties of Pb

Sr. No.	Carbon source	Modified biochar	Method	pH	Temperature	Adsorption capacity (mg g^−1^)	Mechanisms	Advantages	Disadvantages	Ref
1	Crofton weed	MgO-biochar	Impregnation, 60 min	5	600 °C	384.08 mg g^−1^	Mineral precipitation and ion exchange	High adsorption capacity	pH sensitive	43
2	Kiwi branch	KB/Zn–Fe	Pyrolysis		500 °C	161.29 mg g^−1^	Ion exchange, surface precipitation, and complexation	Multiple mechanisms	Leaching risk	80
3	Rice husk	SiBC	Pyrolysis	5	550 °C	230.47 mg g^−1^	Ion exchange, surface precipitation, complexation, and electrostatic adsorption	Structure stability		51
4	Pinus gerardiana	APTES/BC/MXene composite	Sonication	6		323.17 mg g^−1^	Hydrogen bonding, electrostatic interaction, synergistic penetration, and ion exchange	Multifunctional	High-cost synthesis	67
5	Sawdust	Magnetic biochar-iron oxide (MBC-IO) nanocomposite	Pyrolysis	7	500 °C	504 mg g^−1^	Electrostatic attraction, precipitation	Recovery ease	Aggregation occurs	87
6	Macadamia nutshell	FeCl_3_-assisted mechanochemical activation (MCA) biochar	Pyrolysis	<5	400 °C	200.44 mg g^−1^	Interspace filling, electrostatic attractions, cation exchange, and surface precipitation	Highly porous	Needed acidity	61
7	Corn-stalk	Phosphorus-modified corn stover biochar	Pyrolysis	5	550 °C	145.48 mg g^−1^	Precipitation, complexation, and π electron interaction	High affinity	Moderate capacity	53
8	Sewage sludge	SSCB	Co-pyrolysis	6.0	600 °C	152.66 mg g^−1^	Mineral precipitation and ion exchange	Waste reduction	Presence of impurities	81
9	Rice straw	WTR-BC	Co-pyrolysis	3	800 °C	83.8 mg g^−1^	Electrostatic attraction and complexation	Cost-effective	Lower efficiency	52
10	Peanut shells	Nitrogen-doped biochar	Thermal decomposition, 1h	—	350 °C	130.87 mg g^−1^	Complexation, ion-exchange	Enhancement in active sites	Low stability	78
11	Rice husk	MgFe_2_O_4_–NH_2_@sRHB	Carbonization, 450 °C	6.5–7	450 °C	198.93 mg g^−1^	Surface precipitation, electrostatic attraction	Magnetic and functional properties	High cost	39
12	Date leaves and stalks	Multi-functionalized biochar (BC–CO_2_H, BC-EDA, BC-Thioamide)	Pyrolysis, 2 h	2–6	450 °C	61.25 mg g^−1^	Ion exchange, electrostatic cationic attraction	Tunable surface	Low adsorption capacity	85
13	Rice-straw	Nano-hydroxyapatite modified biochar	Calcination, 6 h	5	700 °C	335.88 mg g^−1^	Surface complexation and electrostatic attraction	Strong affinity towards Pb^2+^ ions	CostlyH	77
14	Corn straw	Molybdenum trioxide (MoO_3_)-engineered biochar (MoO_3_-BC)	Pyrolysis, 2 h	4	600 °C	229.87 mg g^−1^	Electrostatic attraction, ion exchange, surface complexation	Highly reactive	Low stability	79
15	Pomelo fruit peel	BC	Carbonization	5	500 °C	92.13 mg g^−1^	Electrostatic attractions, ion-exchange, chemical interaction	Cost-effective and simple	Low capacity	54
16	Pine bark	APTES functionalized nano Fe_3_O_4_ -biochar composite	Pyrolysis	3	500 °C	142.86 mg g^−1^	Ion-exchange, complexation	Reusable ability	Agglomeration occurs	60
17	Peanut shells	Starch-FeS@PSB and Chitosan-FeS@PSB	Pyrolysis, 2 h	5	450 °C	91.74 mg g^−1^, 98.04 mg g^−1^	Complexation, electrostatic attraction, REDOX and physical absorption	Strong binding	Oxidation	65
18	Corn-straw	Calcium-iron layered double hydroxide composite (CaFe-LDH@CSB	Coprecipitation	3.33	600 °C	240.96 mg g^−1^	Surface precipitation	High efficiency	Complex synthesis	63
19	Peanut shell	Biochar	Calcination, 45 min	5	565 °C	2.528 mg g^−1^	Ion exchange and complexation reactions	Cost-effective	Low adsorption capacity	84
20	Earthcare from agriculture waste	Chitosan-modified biochar	Gasification	5		32.23 mg g^−1^	Surface precipitation, ion-exchange	Biocompatible	Low adsorption capacity	64
21	Walnut shell	Chitosan-FeS@WNS	Pyrolysis, 2 h	5	∼250 °C	84.7 mg g^−1^	Electrostatic attraction, hydrogen bonding, physical adsorption, ion exchange, and oxidoreduction	Multifunction	Moderate stability	66
22	Cotton stalk	Biochar	Gasification	5	550 °C	146.78 mg g^−1^	Precipitation, ion exchange, π -π interactions, and complexation	Low cost	Low performance	40
23	Eucalyptus	Magnesium oxide-loaded biochar	Pyrolysis	<4	500 °C	829.11 mg g^−1^	Precipitation and ion exchange	High adsorption capacity	pH dependent	62
24	Cattle manure	Biochar	Pyrolysis	10.84	600 °C	40.8 mg g^−1^	Complexation, precipitation and ion exchange	Cost-effective	Low-efficiency	45
25	Bamboo	(BC)-supported graphene-encapsulated zero-valent iron nanoparticle composites (BC-G@Fe0)	Carbothermal reduction	6	600 °C	62.16 mg g^−1^	Ion-exchange, precipitation	Reductive removal	Stability	103
26	Poplar saw dust	Biochar	Pyrolysis	5	600 °C	62.68 mg g^−1^	Precipitation, ion-exchange, complexation, coordination with π electrons	Simple synthesis	Low adsorption capacity	50
27	Watermelon seeds	Hydrogen-peroxide biochar	Pyrolysis	5	350 °C	60.87 mg g^−1^	Surface complexation	Increase in functional groups	High stability	41
28	Corncob	Mg-modified biochar	Pyrolysis	5.0	450 °C	526.20 mg g^−1^	Surface precipitation, cation π-bonding, complexation, and ion exchange	High adsorption capacity	pH sensitive	38
29	Capsicum annuum L. seeds	CASB	Pyrolysis	5	400 °C	36.43 mg g^−1^	Ion-exchange, surface complexation	Cost-effective	Low adsorption capacity	88
30	T. Angustifolia	CS@S-nZVI/TB w	Chemical reduction	4		281.97 mg g^−1^	Complexation and reduction	High removal rate	Oxidation occurs	104
31	Axonopus compressus	SBC	One-step solvothermal	3	80 °C	191.07 mg g^−1^	Surface precipitation, complexation, ion-exchange	Structural uniformity	High cost	89
32	Olive mill solid waste	BC	Pyrolysis	5	500 °C	40.8 mg g^−1^	Ion exchange and precipitation	Low-cost	Low efficiency	105
33	Maize straw	WBC	Pyrolysis	7–9	500 °C	56.84 mg g^−1^	Surface precipitation, complexation	Simple synthesis	Adsorption capacity decreases	96
34	Larch wood chips	WBC	Pyrolysis-gasification	4.7	485–530 °C	10.2 mg g^−1^	Precipitation and ion exchange	Cost-effective	Low adsorption capacity	102
35	Jackfruit seed waste	JBC	Pyrolysis	7	500 °C	79.9 mg g^−1^	Electrostatic attraction	Simple synthesis		97

### 3.4 Unified mechanistic framework for Pb^2+^ immobilization on functionalized W-BC

Several interactions, such as the incorporation of surface functional groups and the integration of inorganic mineral phases and nanostructured modifiers, explain the adsorption and immobilization of Pb^2+^ ions on W-BC. While studies tend to explain the Pb^2+^ removal through the dominance of a single mechanism, evidence shows that multiple mechanisms are generally active. The extent to which these mechanisms operate is strongly dependent on the feedstock, pyrolysis conditions, solution chemistry, and the employed strategies of surface modification.

The first mechanism is electrostatic attraction, where surface groups such as carboxyl, hydroxyl, and phenolic groups get deprotonated. When they become deprotonated under moderate pH conditions, they develop a negative charge that allows for electrostatic attraction of Pb^2+^. This mechanism primarily accounts for the initial accumulation of Pb^2+^ to the adsorbent surface.

Another mechanism is ion exchange of Pb^2+^ with other naturally existing cationic species, such as Ca^2+^, Mg^2+^, K^+^, and Na^+^, that are present in the mineral fraction of BC. This becomes significant in BCs derived from manure, agricultural, and mineral-rich residues.

Apart from this, Pb^2+^ also undergo surface complexation when they form inner-sphere coordination bonds with oxygen-, nitrogen-, sulfur-, and phosphorus-comprising functional groups. BC functionalization through the incorporation of NM's additives further enhances adsorption capacity and selectivity, as it increasing the number of these binding sites.

In BCs with abundant minerals and metal oxides, surface precipitation plays a significant role. The alkaline oxide's dissolution raises the pH in the immediate vicinity of the adsorbent surface and facilitates the formation of insoluble precipitates such as Pb(OH)_2_, PbCO_3_, Pb_3_(PO_4_)_2_, and PbS. This precipitation strengthens the durability and the diminished metal's mobility.

In some nanocomposites encompasses redox-active components, such as Fe_3_O_4_, MnO_2_, or FeS, additional redox-assisted interactions can also occur. These species modify the electronic interaction at the BC interface and promote the formation of stable Pb-containing phases which also strengthen the holding capacity.

**Fig. 3:**
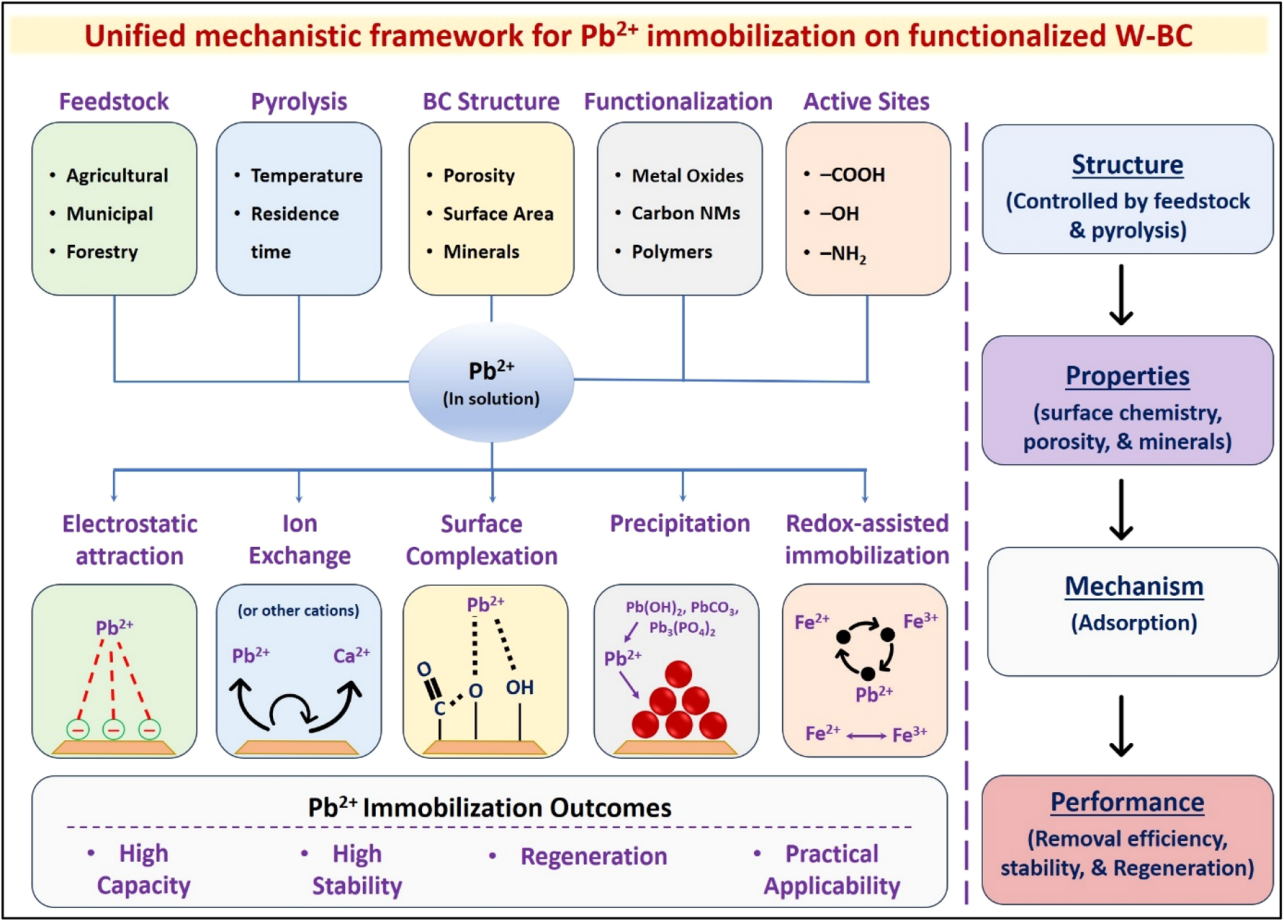
Pictorial representation of unified mechanistic framework for Pb^2+^ immobilization on functionalized W-BC.

Overall, the Pb^2+^ adsorption represent a combination of several contributing factors like electrostatic attraction, ion exchange, surface complexation, precipitation, and redox-assisted immobilization. Fig. 3 shows these mechanisms' predominance is a function of the physicochemical features of BC and the environmental conditions. Thus, future works should go beyond designating a single mechanism and should rather quantify the contributions of several pathways by implementing more sophisticated surface and spectroscopic characterization approaches.

### 3.5 Comparison in the adsorption capacities of raw BC with modified BC

**Fig. 4:**
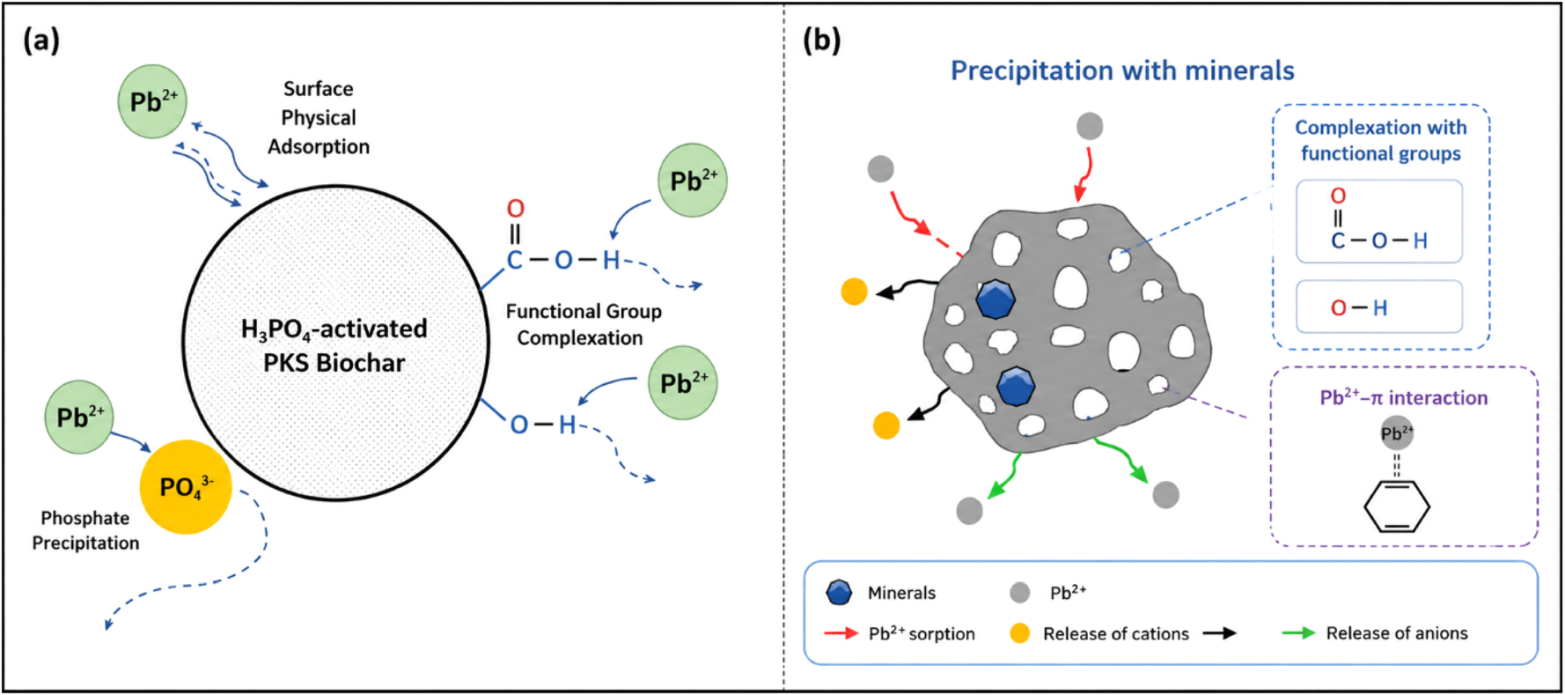
(a) Mechanistic representation of adsorption of Pb(ii) onto the surface of H_3_PO_4_-activated PKS biochar (reproduced from ref. 70 with permission from Elsevier copyright 2023).(b) Adsorption mechanism of Pb^2+^ ions onto the surface of peanut-shell derived biochar (originally drawn by authors).

There are three different ways to modify BC, including physical, chemical, and biological methods, to enhance the adsorption capacity of BC. The chemical modification of biochar mainly includes acid, alkali treatment, and metal impregnation techniques for better adsorption of pollutants. Several studies have reported these modification processes to enhance the efficacy of adsorbents. For instance, Dechapanya *et al.* prepared biochar using palm kernel shells modified with H_3_PO_4_ to improve the adsorbent's efficiency. The study reveals an increase in the porosity of biochar as phosphoric acid decomposes the lignocellulosic materials present in biochar. Moreover, the authors activated the biochar using 0%, 45%, 65%, and 85% H_3_PO_4_ concentration, which, with an increase in concentration, increases the phosphorus and oxygen contents in biochar. The introduction of H_3_PO_4_ introduces PO_4_^3−^ groups, which chemically interact with Pb^2+^ ions *via* a precipitation reaction shown in Fig. 4a.^70^

In another study, Wu *et al.* prepared biochar using peanut shells modified with acidic groups to improve the adsorbent's efficiency. The study includes chemical modification of biochar, in which sulfonation and phosphoric acid activation were performed to increase the functional groups on the biochar surface, helping to improve binding with Pb^2+^ ions. The presence of –SO_3_H groups exhibits strong acidity and ion-exchange capacity. In this context, it is imperative to modify the surface of biochar so that Pb^2+^ is strongly immobilized, reducing the risk of leaching back into the environment.^71^ Furthermore, Wang *et al.*, prepared biochar using peanut shells for the removal of Pb^2+^ ions. The study revealed that at higher temperatures, complexation process reduced. Furthermore, precipitation was the dominant mechanism for the prepared biochar as shown in Fig. 4b.^72^

Also, Qin *et al.* prepared biochar derived from coconut shell. The study utilized oxidative modification of biochar with potassium permanganate and chemical treatment with nitric acid. This modification increases the functional groups on the surface of biochar which improved adsorption efficiency.^73^ Similarly, Liu *et al.* performed chemical modification of prepared biochar with H_3_PO_4_, EDTA, and NaOH to observe the improvement in adsorption of Pb^2+^ ions. Due to highly porous structure prepared from biochar and increase in functional groups, H_3_PO_4_ activated biochar shows improved adsorption performance.^74^ Moreover, Dewage *et al.* prepared biochar was treated with chitosan in acetic acid. The study utilized chemical treatment of the acetic acid-modified biochar with NaOH to introduce amine-based functional groups onto the adsorbent surface. The inclusion of –NH_2_ groups onto the adsorbent enhances coordination between the chitosan amine groups and Pb^2+^ ions.^75^

The study by Liu *et al.* employed combined modification of biochar to improve the adsorption efficiency. Firstly, authors activate the biochar using KOH, then the MgO NPs are utilized as physical treatment. The authors observed that nanoconfinement effect of MgO NPs prevent aggregation, thereby stabilizing the biochar structure.^76^ Various studies have been done using physical, chemical, and biological modification techniques to enhance the stability and efficiency of biochar. On the other hand, several studies reported the enhancement in adsorption capacity in modified BC as compared to pristine W-BC. In 2021, Ahmed *et al.* developed W-BC modified with nitrogen and reported the differences in sorption capacity between unmodified and modified W-BC. The study highlighted that W-BC modified with H_2_O_2_ exhibited enhanced sorption capacities of 25.56 mg g^−1^ and 44.74 mg g^−1^ for HP-BC, compared to bare W-BC.^41^

In 2022, Ahmed *et al.* reported a significant enhancement in adsorption capacity for modified W-BC compared to bare W-BC, with adsorption rates of 63.04 mg g^−1^ for W-BC and 335.88 mg g^−1^ for BC@nHAP.^77^ In another study, Deng *et al.* investigated magnesium-ion-modified W-BC for the adsorption of lead ions and compared its sorption capacity to pristine W-BC. The study revealed that the modified W-BC exhibited a 9.34-fold higher sorption capacity, reaching 448.5 mg g^−1^, compared to bare W-BC.^38^ In the study of removal of Pb^2+^ ion using N-doped W-BC prepared using low-temperature pyrolysis, improved adsorption was found with NBC prepared at 350 °C temperature compared to bare W-BC. The study reported that NBC-350 was five times more effective compared to bare W-BC due to the addition of nitrogen groups to the W-BC. In addition to complexation and ion exchange, the graphitic-N and pyridinic-N were equally effective in the removal of Pb^2+^. It was found that not just physical adsorption, but chemical interaction also works to control the removal of metal ions.

The future research needs improvement in the porosity of W-BC structure to make the process more effective.^78^ The study on the removal of Pb^2+^ derived W-BC from agricultural waste corn straw explored the adsorption capacity of the solution maintained at a pH ≤ 5.08. The study reported that MoO_3_-BC have a higher adsorption capacity of 229.87 mg g^−1^ than pristine BC, which is 5.47 mg g^−1^.^79^ Likewise, Tan *et al.* investigated Zn–Fe-modified BC derived from kiwi branches, and reported that the modified BC exhibited a significantly higher adsorption capacity of 161.29 mg g^−1^, compared to the adsorption capacity of the unmodified BC, which was 36.76 mg g^−1^. The study also shows effect of chloride ions (Cl^−^) enhanced the adsorption capacity, primarily through surface precipitation and ion exchange mechanisms.^80^

The study of Wang *et al.* reported the synthesis of BC derived from sewage sludge modified with the CaSO_4_ to improve its adsorption capacity. The presence of calcium sulfate/gypsum react with C to generate more of the CaS on the surface of BC which enhance the removal of Pb^2+^ ions through ion exchange and mineral precipitation.^81^ The ion-exchange and the mineral precipitation are the important adsorption mechanism for the Pb^2+^ removal. The study observed that the adsorption capacity of sewage sludge-derived BC doped with silica increases upto 230.47 mg g^−1^, which reported that it is 6.43 times higher than that of the bare BC. This significant enhancement in adsorption capacity can be attributed to the increase in surface area and porosity caused by the incorporation of silica.^51^ In 2024, Ha *et al.* investigated ferrous-modified BC to enhance the adsorption efficacy of metal ions. The study found that BC loaded with Fe_3_O_4_ has three times greater adsorption capacity than rice husk-derived BC.^82^ Likewise, Subramanian *et al.* compared the performance of acid-modified BC with pristine BC to evaluate the adsorption efficacy of Pb^2+^ ions. The study found that the highest removal potential of nitric acid-modified W-BC compared to the both bare BC and phosphoric-loaded BC. The adsorption capacities for Prosopis juliflora-derived BC, phosphoric, and nitric-acid loaded BC were 10.98, 17.32, and 18.72 mg g^−1^ respectively.^83^

Although many studies highlight BC's use for the effective removal of Pb^2+^ ions, the primary mechanisms remain poorly understood. Many studies report precipitation as Pb^2+^ removal mechanisms in Ca, Mg, and phosphate containing species. Conversely, surface complexation removes Pb^2+^ in BCs with functional groups containing oxygen (O), hydroxyl (OH), and carboxyl groups (C=O). These classic and subtle Pb^2+^ binding mechanisms can be attributed to many aspects such as BC's feedstock composition, pyrolysis temperature, solvent-based chemistry, and BC's modification methods. For example, precipitation and ion exchange pathways will be favoured when BC is produced at high temperatures due to the increased aromaticity and mineral content. In contrast, low-temperature BCs will have retained more O-containing functional groups, which favour surface complexation. Precipitation-dominated BC adsorption exhibits due to local effects of alkalization, but in case of carbon-based nanocomposites as adsorbents, the adsorption occurs due to the interactions dominated by coordination and electrostatic effects. It concludes that the Pb^2+^ adsorption mechanisms are numerous and are best characterized as a synergistic process. But the lack of common methods for characterization and mechanistic evaluation of adsorbents is a major hindrance that prevents the development of consistent models.

## 4. Experimental parameter

The parameters that govern Pb^2+^ adsorption must be considered in the context of the physicochemical attributes of the synthesized BC. The adsorbent behavior evaluated as a function of pH, adsorbent dosage, and contact times reflects the BC's disposition and structural traits stemming from its prior synthesis and functionalization.

In this section, we discuss various parameters that influence the adsorption efficiency of metal ions, with a focus on the pH, concentration of the adsorbent.

### 4.1 Effect of pH

The pH of the solution controls both the speciation of metal ions and the surface charge of the adsorbent, making it a crucial factor in adsorption processes. According to numerous research, removal efficiency often rises as pH rises. This is usually due to the reduction of protons of functional groups on an adsorbent surface, which becomes more negatively charged. For instance, Li *et al.* observed that the adsorption capacity of Pb^2+^ increased as the pH increased from 2.33 to 8.66, with the sorption capacity rises from 45.8 mg g^−1^ to 50 mg g^−1^. The study revealed that almost all Pb^2+^ was removed from wastewater when the pH exceeded from 4, highlighting the effect of pH in enhancing adsorption efficiency.^79^ The possible surface reaction is as follows:–COOH + Pb^2+^ ⇌ COO^−^ Pb^2+^ + H^+^In another study, Dinh *et al.* reported the highest adsorption efficiency at pH 5. When pH of the solution increases, the surface of BC becomes negatively charged due to the deprotonation of functional groups, enhancing electrostatic attraction between the BC surface and positively charged metal ions. This increase in electrostatic attraction improves the adsorption efficiency of metal ions.^54^ In contrast, at lower pH values, the adsorption efficiency was reduced due to competition for adsorption sites between the positively charged BC surface and the metal ions, both of which carry a positive charge. Likewise, Puglla *et al.* studied the pH effect on the adsorption of Pb^2+^ ions and reported that as pH increases from 3 to 5, the rise in adsorption efficiency was seen due to electrostatic attraction between metal ions and adsorbent. However, as pH increases more from 7 to 9, the adsorption efficiency began to decrease due to complex formation that decreases the concentration of Pb^2+^ ions. At pH > 7, the formation of hydroxide (Pb(OH)_2_, Pb(OH)_3_^−^ ) complexes takes place, which are anionic and do not adsorb on the surface of BC, leading to a decrease in adsorption potential.^84^Pb^2+^ + 2OH^−^ → Pb (OH)_2_(s)

This is because BC typically adsorbs cationic species and the formed complexes Pb(OH)_3_^−^ and Pb(OH)_4_^2−^ are anionic. Similarly, Shi *et al.* studied the pH effect on the adsorption capacity of BC and observed that at pH values less than 6, Pb^2+^ is the dominant species in the solution. However, at pH values above 6, the formation of Pb^2+^-containing complexes begins to occur. The removal efficiency was found to be higher at lower pH values due to the increased concentration of H^+^ ions, which protonate the surface of the adsorbent.^62^ In the same way, Zahedifar *et al.* found that when the pH of BC-thioamide was close to 3.5, the adsorbent surface became negatively charged and electrostatic interactions gets stronger.^85^ The authors suggested that the optimal pH for their study was 5, at which the interactions were stronger and the adsorption efficiency was highest. Likewise, in the study of Pb^2+^ ion adsorption, it was found that as the pH of the solution increased, the adsorption potency also increased up to a certain point. However, with further increases in pH, the formation of complexes between Pb^2+^ ions and the functional groups on the BC surface began, leading to a decrease in adsorption efficiency.^51^ Also, Zhou *et al.* revealed an optimum pH of 5 for achieving the highest adsorption of Pb^2+^ ions.^53^ Most of these studies revealed an increase in adsorption efficiency in the range of pH 4–6, beyond which a decline is observed. At higher pH values from (>6–7), formation of hydroxide complexes such as Pb(OH)_2_, Pb(OH)_3_, and Pb(OH)_4_^2−^ occurs. These complexes decrease the concentration of Pb^2+^ ions and in some cases, it generates anions that do not adsorb to the negative charged biochar. As such, adsorption efficiency either remains constant or falls above the optimum pH value. The observed trend indicates that pH∼5 are most favorable for Pb^2+^ adsorption due to the balance between surface charge and metal ion availability.

### 4.2 Effect of dosage of adsorbent

The concentration of the adsorbent is one of the important parameters that plays momentous role in the adsorption process of BC. Several studies have shown that as the adsorbent dosage increases, the availability of active sites for metal ions also increases, thereby enhancing the adsorption capacity. A higher concentration of adsorbent provides more surface area and active binding sites, allowing for greater interaction with the metal ions and improving the overall removal efficiency. However, beyond a certain point, a saturation effect occurs, where further increases in adsorbent concentration may Pb^2+^ to a decrease in adsorption efficiency. This is due to factors such as aggregation of the adsorbent particles, which reduces the available surface area for effective adsorption. For instance, Shi *et al.* reported the effect of the concentration of BC, which shows that increasing the concentration of BC was favorable to increase the adsorption efficacy.^62^ In another study, Puglla *et al.* investigated the adsorption efficiency of Pb^2+^ ions by optimizing the concentration of BC. The study found that at a concentration of 12 g L^−1^, 74% removal of Pb^2+^ ions was achieved. Increasing the BC concentration to 14 g L^−1^ resulted in an 85% removal efficiency. However, once equilibrium was reached, further increases in BC concentration did not Pb^2+^ to a higher removal percentage, indicating that the adsorption sites became saturated and no additional Pb^2+^ ions could be adsorbed, regardless of the BC amount used.^84^ Likewise, Zahedifar *et al.* found that increasing the adsorbent dosage significantly improved removal efficiency. The authors examine the effect of adsorbent dosage from 5 to 30 mg, the high absorption was reached with 20 mg of adsorbent due to an increase in active sites and functional groups.^85^ Another work by Wang *et al.* evaluates the increase in removal potential with an increase in BC dose but simultaneously a decrease in adsorption capacity from 112.5 ± 1.6 mg g^−1^ to 46.5 ± 2.3 mg g^−81^. Comparative studies indicate that optimal dosages vary depending on biochar type and modification. The general trend confirms that there exists a threshold dosage beyond which no significant enhancement occurs.

### 4.3 Effect of contact time

The Contact time determines the time how long the adsorbent interacts with the metal ions in solution. Several studies reported that adsorption efficiency increases initially with contact time due to more available sites and the diffusion of metal ions into the pores of BC and forms stable bonds. However, the rate slows down as the active sites become occupied, eventually reaching an equilibrium where no further significant increase in removal efficiency occurs. For instance, Zahedifar *et al.* reported that initially, 98% of Pb^2+^ was removed from the aqueous solution within the first 120 minutes, owing to the presence of vacant adsorption sites on the surface of BC-Thioamide/MNPs. However, as contact time increased, desorption of the adsorbed species began to occur, leading to a decrease in removal efficiency.^85^ In another study, Dinh *et al.* assessed the impact of varying contact time on the adsorption efficacy over a range of 5 to 240 minutes. A rapid increase in adsorption was observed during the first 60 minutes. However, after 120 minutes, all the active sites were saturated, and the removal efficiency stabilized, with little to no further increase in adsorption observed.^54^ Likewise, Ahmad *et al.* observed the rise in Pb^2+^ adsorption in the initial 50–60 min reveals the occupation of active sites which slowly decreases with time. The study reported the equilibrium after 800 and 500 min which shows the phenomenon of chemisorption.^41^ Overall, it founds that in several studies' equilibrium reached within 60–120 minutes, although some of the studies indicates that their material requires long time depending on biochar properties and experimental conditions. The studies exhibit that chemisorption mechanism is dominant mechanism which involves strong interactions such as surface complexation and ion exchange. After equilibrium is reached, no significant increase in adsorption occurs, and in some cases, slight desorption may be observed due to system instability. The summary of adsorption parameters for Pb^2+^ ions has been illustrated in Table 4.

**Table 4:** Summary of adsorption parameters of Pb^2+^ ions using biochar

Sr. no.	Material	pH	Adsorbent dosage	Contact time	Mechanism	Removal rate	Reference
1	BC-thioamide	5	20 mg	120 min	Chemisorption	∼98%	106
2	Biochar	5–7	14 g L^−1^	45 min	Physisorption	95.96%	84
3	Biochar	6	1 g L^−1^	6 h	Mineral precipitation and ion exchange	96.2%	81
4	Biochar	5	—	120 min	Ion exchange, electrostatic interactions	70–80%	54
5	MoO_3_-BC	4	0.5 g L^−1^	240 min		99.9%	79

### 4.4 SPM–performance relationship

As there are many investigations on the ability of various W–BCs to adsorb Pb^2+^, it is equally important to consider the BC's synthesis and characteristic features in the context of its adsorption ability. An understanding of the SPM–performance of BCs is imperative in this regard.

The first controlling factors of adsorption performance is indicated by the feedstock selection. Cellulose and hemicellulose are abundant in agricultural residues, and following pyrolysis, BCs are formed with a porous structure. Alternatively, BCs derived from animal manure are enriched with mineral species, such as Ca, Mg, and P, and therefore contain precipitates and ions that facilitate exchange. Therefore, the feedstock composition will determine the predominant pathway of Pb^2+^ immobilization.

The choice of pyrolysis conditions will also determine the BC's structure and chemistry. BCs differ based on the temperature. For instance, low temperature BCs retain a higher density of oxygen-based functional groups. These facilitate complexation of Pb^2+^ on the surface. High temperatures will increase the development of minerals, porosity, and aromaticity, which will favour the ion-exchange processes and precipitation. The development of a porous structure will collapse at excessive temperatures and functional groups will be reduced causing a negative impact on adsorption.

NM's functionalization provides more control over their adsorption properties. The use of metal oxides like MgO, Fe_3_O_4_, and MnO_2_ as modifiers can lead to an increase in the hydroxyl's density and active mineral sites, which can notably increase the potential for precipitation and surface complexation. Carbon-based NMs mainly enhance the surface area and facilitate electron transfer. In comparison, the polymer-based modifiers contain functional groups that can organize favourable interactions with Pb^2+^ ions through coordination.

The traits like surface area, porosity, mineral composition, surface charge, and the density of functional groups, help to describe the specific contributions of mechanism, including ion-exchange, surface complexation, precipitation, and the redox-assisted immobilization. For this reason, it is essential to consider all the functionalization effects when evaluating the adsorption performance as they build from the feedstock selection and fabrication conditions. It is important to establish cause–effect relationships for a balanced design of novel BC adsorbents and to ensure that comparisons of the literature is valid.

## 5. Regeneration/reusability study

Regeneration/*reusability* is an important parameter considered during the practical applications of BC for removal of metal ions. Various studies reported BC as an effective adsorbent to treat wastewater but it is important to assess the regeneration potential of prepared BC for its long-term applications. To ensure the cost-effectiveness and sustainability of BC, it is important to conduct a regeneration study. The regeneration and desorption study involves the chelating agents, saline solutions, and acids as desorption agents to check their recyclability. Various researchers have worked on desorption studies to assess the potential of BC for regeneration. For instance, Wang *et al.* reported the five adsorption/desorption cycles with the adsorption efficiency decreased from 90.62% in the first cycle to 70.56% by fifth cycle. The study observed that despite of reduction in efficiency, the regenerated W-BC still shows strong absorptivity towards Pb^2+^ ions, indicating its potential for the removal of Pb^2+81^. Likewise, Zhou *et al.* reported the extraction of MCA-BC using an HNO_3_ solution to assess the reuse potential of the prepared material. The study found that the removal rate of contaminants still reached 70% after multiple reuse cycles.^86^ To observed the reusability of BC in water treatment, the study conducted desorption study which revealed that even after three cycles the BC shows 80% removal efficiency.^87^ Hammo *et al.* found reduction from 51.75 to 14.58% in the adsorption capacity from the first cycle due to degradation of structure caused due to acids utilized in study.^88^ Likewise, Yu *et al.* were able to achieve 65% of removal rate even after 5 cycles using HNO_3_ as desorbent. The study reported that after reusing HNO_3_ for multiple times to remove the metal ions, the structure of BC started break down into smaller pieces which further enhances the adsorption capacity of BC attributed to enhancement in better interaction with metal ions, and surface area.^89^ The study of Wu *et al.*, reported the sorption ability of BC at 271.53 mg g^−1^ even after 5 consecutive cycles with usage of NaOH as an eluent.^90^ Likewise, Zhang *et al.* reported high adsorption capacity of CoFe_2_O_4_@PBC-LDH that was 83.14 mg g^−1^ even after seven cycles.^91^ Several studies utilized different desorbents including NaNO_3_, Na_2_EDTA, HNO_3_, CaCl_2_, NaOH, HCl. And KNO_3_ for the desorption studies with varying concentrations. Some studies have shown the effective adsorption of Pb^2+^ ions even after multiple cycles of reusing, likely due to presence of surface functional groups, and stable structure of BC which works as reusable nano-structured remediation material. However, other studies reported a decrease in the adsorption capacity of BC which happens due to deactivation of functional groups, gradual saturation of adsorption sites, and absence of active functional groups. The regeneration ability of W-BC is strongly aligned with the sustainable green chemistry principles. Among twelve green chemistry principles, W-BC supports waste prevention, catalysis, the use of renewable feedstocks, designing safer chemicals, energy efficiency, and the use of less hazardous chemical synthesis. In addition to this, the incorporation of magnetic materials enhances their separation, which reduces the secondary waste products generation and promotes practical scalability. The comparison among different recycling cycles shown in Table 5. Overall, there is an urgent need for further research to identify sustainable methods for BC regeneration, optimize desorption efficiency, and enhance its long-term effectiveness for environmental applications.

**Table 5:** Comparison of reusability and removal efficiency of different W-BC

Sr. no.	Biochar	Desorbent	Adsorption/Desorption cycles	Removal rate after reuse	References
1	SSCB	Na_2_EDTA	5	70.56 ± 4.56%	81
2	MCA-BC	HNO_3_	5	70%	86
3	MBC-IO	HCl	3	80%	87
4	CAS-BC	EDTA, HCl, and HNO_3_	5	14.58%	88
5	S-BC	HNO_3_	5	65%	89
6	PSD-B			72.42%	50
7	APTES/BC/MXene	NaNO_3_	5	96.2%	67
8	NBC-350-0.1	NaOH	5	80%	78
9	CS-BC	HNO_3_	3	62.6%	40
10	P-BC	HNO_3_	5	72.88%	54
11	CaFe-LDH@CSB	EDTA		71.28%	63

### 5.1 Toxicity and post-adsorption fate of lead-loaded biochar

It is critical to observe the long-term stability and toxicity of lead-loaded biochar after adsorption. The environmental remediation is the most important factor in which real-world applications of prepared adsorbents are considered. Therefore, it is urgent to evaluate the stability of immobilized toxic metals under several environmental conditions. Nowadays, biochar exhibits a promising solution for the adsorption of toxic metals from the environment, but assessing its post-adsorption fate is still a big challenge in real-world scenarios. Several studies have explored the toxicity and post-adsorption fate of Pb-loaded biochar, revealing their impact on metal leaching potential and overall environmental risk. For instance, Liu *et al.* observed the toxicological effect of lead-loaded biochar on *Escherichia coli*. The study revealed that biochar prepared at 700 °C inhibited the growth of *E. coli*, which was attributed to the release of Pb^2+^ from the biochar. At higher concentrations, biochar aged with Pb^2+^ led to an increased bioavailability of Pb^2+^.^92^

## 6. Statistical analysis

**Fig. 5:**
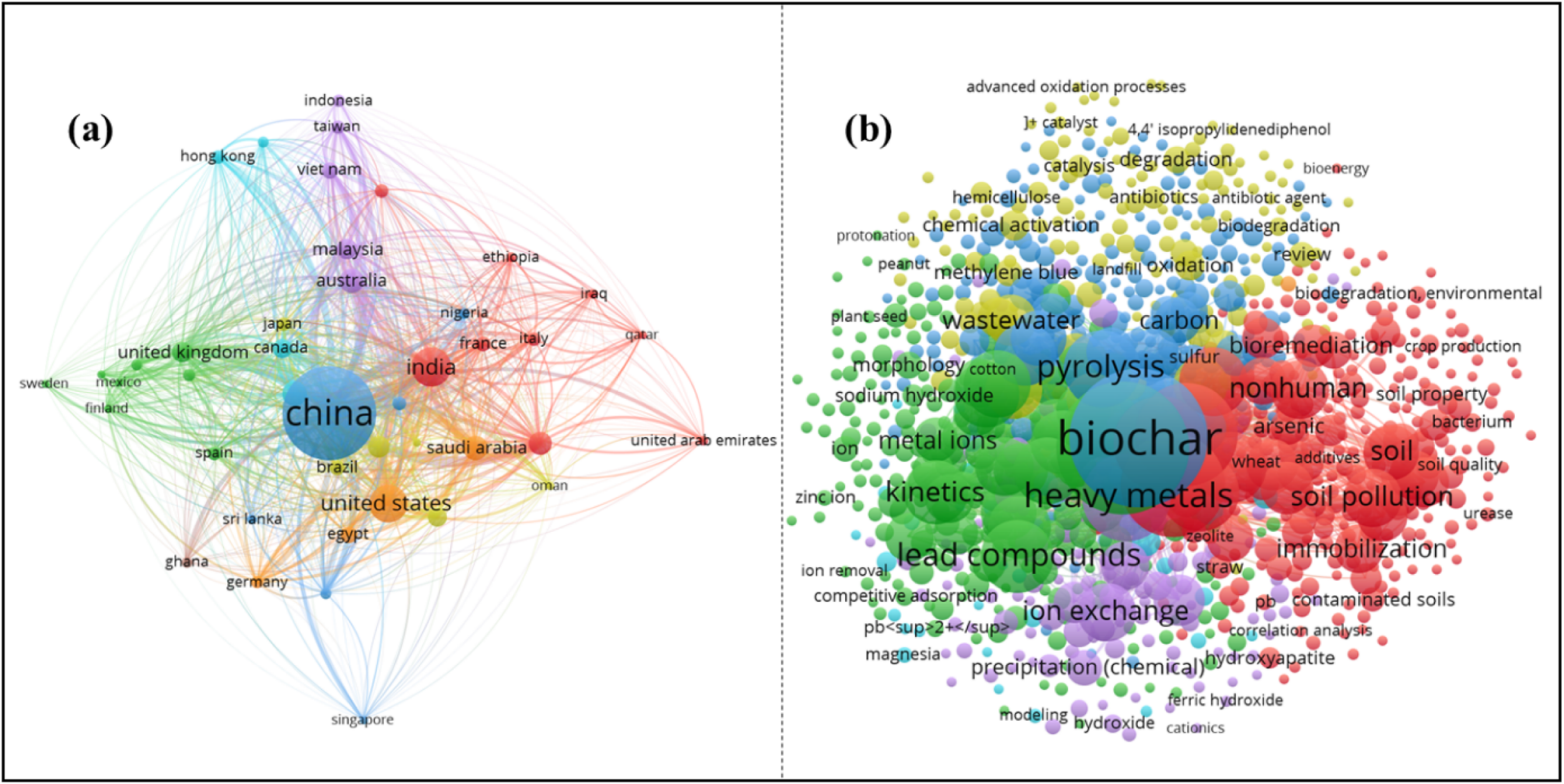
(a) Country-wise, (b) keywords data collected from VOS viewer.

The statistical analysis using VOSViewer data has been done by collecting CSV files from SCOPUS using an advanced search query string, TITLE-ABS-KEY (“Biochar” AND “Adsorption” AND “lead”) AND PUBYEAR %3e 2018 AND PUBYEAR %3c 2026 AND PUBYEAR %3e 2019 AND PUBYEAR %3c 2026 AND (LIMIT-TO (LANGUAGE, “English”)). Fig. 5 represents country-wise, and keywords data extracted from Scopus have been shown in the graphical representation using VOSViewer software. Fig. 5a reveals the country-wise research on the adsorption of Pb^2+^ using biochar. The analysis on country-wise research shows that China implies higher productivity, while thicker links shown the active international research collaborations. The clusters represent the group of countries which have more research collaboration opportunities. On the other hand, weaker link strength indicates a lack of international cooperation between certain countries. Fig. 5b represents the keyword co-occurrence network with main research themes and hotspots. In general, keywords like “biochar,” “adsorption,” and “lead” form the network's backbone due to their central location in the diagram, and they also point to their importance in this topic. Clusters are observed, and each cluster includes particular keywords that characterize some aspects of biochar lead absorption: adsorption processes, biochar modification techniques, heavy metal removal processes, and wastewater treatment uses, *etc.* Nodes with big sizes represent already researched issues, while smaller nodes refer to novel research topics.

Keyword clusters interconnect to illustrate interdisciplinary characteristics of the area under consideration: it encompasses environmental science, materials engineering, chemistry. Also, the distribution of keywords indicates that research focus shifts from theoretical adsorption process study to more applied research, such as wastewater treatment and new materials creation.

## 7. Future directions

Although numerous efforts have gone into the use of W-BC for Pb^2+^ removal, many hurdles in science and engineering remain before this research can be effectively used. In general, no standardization for analytical methods and synthesis protocols have hindered this research. Apparent inconsistency in reported adsorption mechanisms and performance is mainly due to the varying compositions of the precursor material, conditions used for pyrolysis, chemistry of the solutions used in the experiments, and the design of the experiments themselves.

Mechanistic ambiguity presents another significant issue. Pb^2+^ removal is ascribed to electrostatic attraction, ion exchange, surface complexation, precipitation, and redox-assisted immobilization. However, the degree of each pathway remains largely unknown. Most studies rely on indirect evidence from adsorption experiments, while *in situ* characterization techniques, such as X-ray absorption spectroscopy (XAS) and synchrotron-based techniques, *operando* FTIR spectroscopy, and solid-state NMR, have been underused. Future work should emphasize the mechanistic quantification of Pb^2+^ removal, adsorption and immobilization pathways.

Most current studies are conducted under ideal lab conditions by utilizing the single-component solutions. In reality, wastewater contains many components, including many different ions, organic matter, and varying pH. All of these can have a major impact on the adsorption process. For this reason, systematic research on multi-component solutions and real wastewater is required to understand the selectivity and utility of the system.

Further, environmental stability is a long-term concern, as most studies only report the maximum adsorption capacity and do not consider what happens to BC in the long-term exposure of Pb. Many environmental changes like aging, microbial actions, pH alterations, and oxidation, will affect the stability of immobilization and may cause secondary release of Pb ions. Therefore, studies on long-term stability and leaching behaviour of BC must be conducted.

**Fig. 6:**
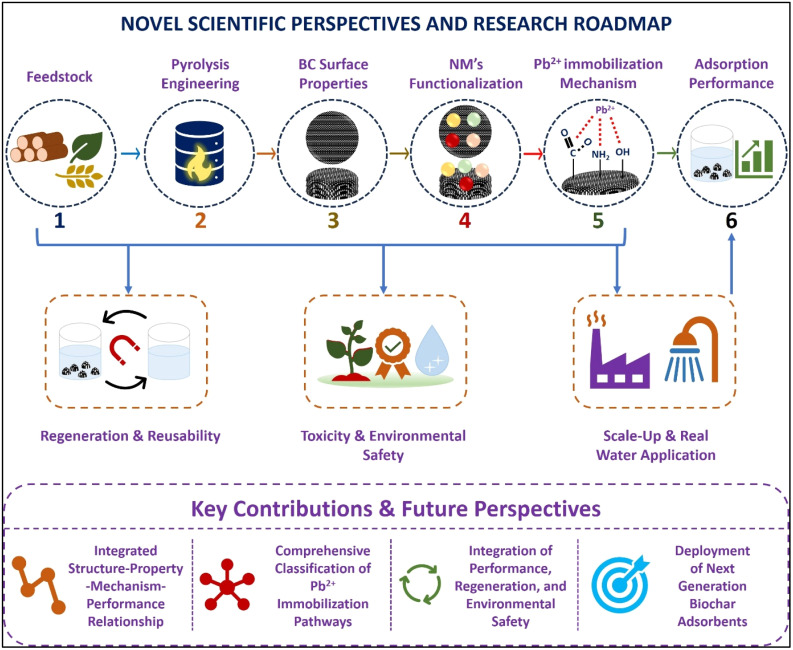
Pictorial illustration of scientific perspectives for future use of Pb^2+^ removal using W-BC systems.

Till now, very few regeneration studies have been conducted. Most studies evaluate less than 5 adsorption–desorption cycles, which does not give a clear understanding of the structural deterioration, active site's spoilage, and the potential costs. Future studies should aim to create a sustainable standard for regeneration. In addition, it is also important to note the challenges faced while transferring laboratory-scale experiments to practical deployment. The studies conducted recently have neglected the validation of pilot-scale studies, life-cycle assessments, techno-economic analyses, integration of processes, and compliance with regulations. The further development of these studies will be critical in determining the feasibility of the laboratory results that can be implemented in large-scale wastewater treatment. A pictorial demonstrations and details of novel scientific perspective for future use W-BC to remove Pb^2+^ are attached in Fig. 6 and Table 6, respectively.

**Table 6:** Priority research directions for next-generation waste-derived biochar systems

Challenge	Current limitation	Future research priority
Mechanistic understanding	Mechanisms inferred indirectly	*Operando* spectroscopy and XAS studies
Standardization	Different synthesis conditions	Universal evaluation protocols
Selectivity	Single-ion systems dominate	Multi-ion wastewater studies
Stability	Limited aging studies	Long-term leaching assessment
Regeneration	≤5 cycles in most studies	Extended cyclic durability studies
Scale-up	Laboratory-scale focus	Pilot-scale demonstration
Sustainability	Limited economic analysis	Life-cycle and techno-economic assessment

Briefly, the laboratory studies have to focus on providing pilot studies, real wastewater testing, quantitative mechanistic investigations, and long-term stability assessments. Addressing these issues will allow the design of new BC sorbents with their enhanced activity, and allow their implementation for the Pb ions remediation in a sustainable manner.

## 8. Conclusions

This review introduces a novel concept of a consolidated framework that interconnects feedstock choice, synthesis parameters, nanomaterial functionalization, adsorption mechanisms, and Pb^2+^ removal. Unlike, previous reviews that summarize only the adsorption capacity data, this one describes the structure–property–mechanism relationships of Pb^2+^ immobilization, and explains how surface chemistry and mineralogy dictate adsorption mechanisms. This review integrates the mechanistic, regeneration, and toxicity paradigms with the logistical and operational challenges, to advance the design of novel W-BC systems for sustainable Pb^2+^ removal.

This review provides a comprehensive analysis on the recent development on the adsorption of Pb^2+^ ions using W-BC, focusing on articles published between 2020-2024, particularly regarding the use of different natural feedstocks for Pb^2+^ removal. This review explores various processes utilized for contaminant removal, with adsorption emerging as the most preferable process. Furthermore, a comparative evaluation of reported adsorbents highlights that adsorption capacities of bare BC are low compared to modified BC, as modification with NMs enhances the surface area and functional groups present on the surface of BC. Also, the variation in adsorption capacities ranging from as low as 32.23 mg g^−1^^64^ to as high as 504 mg g^−1^^87^ is attributed to differences in synthesis conditions, surface modification strategies, and operational parameters. The study also highlights the regeneration process and mechanisms during the adsorption of heavy metal ion. Moreover, modification with magnetic materials has shown success in regeneration due to the ease of separation.

Overall, this review shows that the adsorption performance of W-BC heavily relies upon the development history and the surface properties. Writing this review established some links between the preparation conditions, the adsorption mechanisms and the performance of the material. This is the first step to the rational design of subsequent biochar derived adsorbents. The analysis and the mechanism that was presented in this review are expected to help the sustainable Pb^2+^ remediation and the wastewater treatment.

## Conflicts of interest

The author(s) declare(s) that there is no conflict of interest regarding the publication of this article.

## Data Availability

Data sharing not applicable to this article as no datasets were generated or analysed during the current study. Supplementary information (SI) is available. See DOI: https://doi.org/10.1039/d6ra02626e.
